# Synthetic Gene Circuit-Based Assay with Multilevel Switch Enables Background-Free and Absolute Quantification of Circulating Tumor DNA

**DOI:** 10.34133/research.0217

**Published:** 2023-10-02

**Authors:** Chao Zhang, Zedong Li, Jie Liu, Chang Liu, Haoqing Zhang, Won Gu Lee, Chunyan Yao, Hui Guo, Feng Xu

**Affiliations:** ^1^ The Key Laboratory of Biomedical Information Engineering of Ministry of Education, School of Life Science and Technology, Xi’an Jiaotong University, Xi’an 710049, P.R. China.; ^2^ Bioinspired Engineering and Biomechanics Center (BEBC), Xi'an Jiaotong University, Xi’an 710049, P.R. China.; ^3^ TFX Group-Xi'an Jiaotong University Institute of Life Health, Xi'an 710049, P.R. China.; ^4^ Department of Mechanical Engineering, Kyung Hee University, Yongin 17104, Republic of Korea.; ^5^ Department of Transfusion Medicine, Southwest Hospital, Third Military Medical University (Army Medical University), Chongqing 400038, P.R. China.; ^6^ Department of Medical Oncology, The First Affiliated Hospital of Xi'an Jiaotong University, Xi'an 710061, P.R. China.

## Abstract

Circulating tumor DNA (ctDNA) detection has found widespread applications in tumor diagnostics and treatment, where the key is to obtain accurate quantification of ctDNA. However, this remains challenging due to the issue of background noise associated with existing assays. In this work, we developed a synthetic gene circuit-based assay with multilevel switch (termed CATCH) for background-free and absolute quantification of ctDNA. The multilevel switch combining a small transcription activating RNA and a toehold switch was designed to simultaneously regulate transcription and translation processes in gene circuit to eliminate background noise. Moreover, such a multilevel switch-based gene circuit was integrated with a Cas9 nickase H840A (Cas9n) recognizer and a molecular beacon reporter to form CATCH for ctDNA detection. The CATCH can be implemented in one-pot reaction at 35 °C with virtually no background noise, and achieve robust absolute quantification of ctDNA when integrated with a digital chip (i.e., digital CATCH). Finally, we validated the clinical capability of CATCH by detecting drug-resistant ctDNA mutations from the plasma of 76 non–small cell lung cancer (NSCLC) patients, showing satisfying clinical sensitivity and specificity. We envision that the simple and robust CATCH would be a powerful tool for next-generation ctDNA detection.

## Introduction

Circulating tumor DNA (ctDNA) is highly associated with tumor development and contains almost complete tumor genetic information [1], which has enabled rapid advances in dynamic assessment of cancer treatment [2,3], due to its easily accessible sampling and high specificity to tumor cells. However, the concentration of ctDNA is ultralow in biological fluids and ctDNA is highly diluted with abundant cell-free DNA (cfDNA) from somatic cells, with a 1-cm^3^ tumor only producing 0.001 to 0.03% of ctDNA in total cfDNA [4]. Meanwhile, ctDNA concentration varies by orders of magnitude, especially in the ctDNA mutation rate [5]. Thus, highly sensitive and specific technologies with large dynamic range are expected to meet the precise detection requirement of ctDNA in clinics.

Clinically, next-generation sequencing (NGS) and quantitative polymerase chain reaction (qPCR) have been widely used for ctDNA detection. NGS shows a satisfying detection range, but it is technically demanding and time-consuming, and shows discordant reproducibility in ctDNA detection [6–8]. qPCR exhibits decent rapidness and sensitivity, but it is associated with the issues of dedicated thermocycling protocol for each assay, possible contamination from the amplified template, and relative quantification relying on standard calibration [9]. Alternatively, the assays based on isothermal amplifications [e.g., recombinase polymerase amplification (RPA)] have been developed with their simple operation and competitive sensitivity [10]. However, these assays are based on exponential amplifications, which usually introduce inevitable background noise [11–13]. Background noise in nucleic acid detection mainly refers to unwanted or extraneous signals caused by nonspecific amplification or contamination during the detection process, which will introduce false-positive signals to assays that rely on end-point signal readout [14–17], thus resulting in erratic detection sensitivity, specificity, and dynamic range, posing a critical bottleneck for precise ctDNA detection. Hence, it is crucial to develop new background-free assays for ctDNA detection.

Some important efforts have been devoted to eliminating background noise [16,18–21], focusing on developing alternative reporters for improving signal-to-noise ratio (SNR) or suppressing unspecific signal. For example, the background autofluorescence as well as photon scattering interference could be eliminated by using near-infrared upconversion nanoparticles as reporter [19,22,23]. Although these assays could enhance the positive signal, unspecific target amplification still exists, leading to undesirable background noise. Alternatively, functional nucleic acids have been exploited to improve the target recognition specificity, resulting in significantly decreased unspecific signal [24,25]. For instance, short oligonucleotides with topologic structure were designed to avoid nonspecific amplifications [16]. Although these assays can effectively reduce background noise, the introduction of additional unspecific signal absorption lengthens the whole detection time (>3 h) [26].

Lately, the state-of-the-art tools offered by synthetic biology have enabled the construction of gene circuit-based assays with customizable performance [27–30], which are typically constituted by recognizer, gene circuit, and reporter [31]. For nucleic acid detection, target nucleic acids are directly identified by the recognizer, then activate the following gene circuit, and finally produce readable signal with the reporter. Upon receiving an input, the gene circuit is activated to produce proteins as an output through transcriptional and translational processes that are regulated by switches. The switch plays a pivotal role in gene circuit by precisely regulating the signal output level, which decides the background signal, SNR, and dynamic range [13,32–35].

**Fig. 1.**
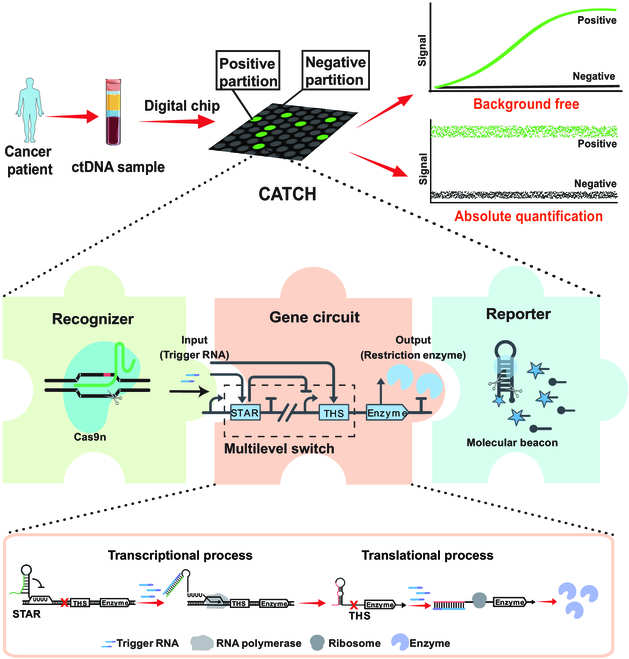
Scheme of synthetic gene circuit-based assay with multilevel switch enabling background-free and absolute quantification of ctDNA. A multilevel switch is designed to simultaneously regulate transcription and translation processes to reduce background noise. Cas9n as the recognizer and MB as the reporter are integrated with the gene circuit to form CATCH. CATCH can be further integrated with a digital chip to provide background-free and absolute quantification of ctDNA.

Inspired by the excellent controllability of gene circuit, here, we developed a synthetic gene circuit-based assay with multilevel switch (termed CATCH) for background-free and absolute quantification of ctDNA (Fig. 1). More specifically, we developed a multilevel switch and used it in gene circuit to avoid background noise. The multilevel switch simultaneously regulates the transcription and translation in the gene circuit relying on a small transcription activating RNA (STAR) and a toehold switch (THS), respectively. Moreover, we used Cas9 nickase H840A (Cas9n) as the recognizer and molecular beacon (MB) by restriction enzyme digestion as the reporter, which are integrated with the multilevel switch-based gene circuit to form CATCH. We demonstrated that CATCH can be done in one pot to prototype ctDNA detection, showing virtually no background signal when no target DNA is present, and achieve robust absolute quantification of ctDNA when integrated with a digital chip. Further, we demonstrated the clinical application of CATCH for detecting ctDNA from non–small cell lung cancer (NSCLC) patients’ plasma. We envision that the simple and robust CATCH would be a powerful tool for next-generation ctDNA detection.

## Results

### Multilevel switch combining STAR and THS is designed for background-free output in gene circuit

**Fig. 2.**
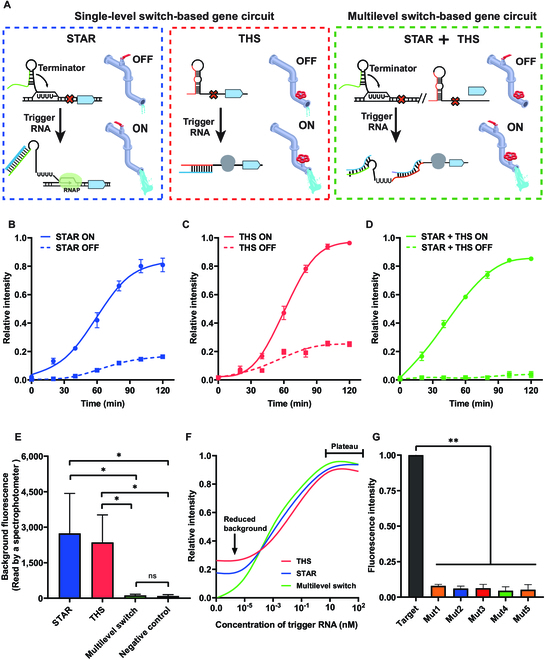
The multilevel switch enables background-free output of the gene circuit. (A) The scheme shows single-level and multilevel switches. The multilevel switch uses 2 separate switches in series, which simultaneously regulates the transcriptional and translational processes. The water pipe serves as a representation of the signal output process in the genetic circuit, where both STAR and THS can be likened to the valves in the water pipe. In the absence of a target, a small amount of water continues to flow, indicating the generation of background signal. However, the implementation of a multilevel switch ensures the absence of background signal. (B to D) Time-dependent fluorescence signal changes are determined from gene circuit based on STAR (B), THS (C), and multilevel switch (D) for 0 and 1 pM of synthetic trigger RNA detection. (E) The background noise of different switch-based gene circuits is calculated when no target RNA is present. (F) Dose responses of different gene circuits show that the multilevel switch can efficiently reduce the background output while maintaining the maximum output levels. (G) We designed 1 to 5 mutations in trigger RNA. The fluorescence response produced by single-base mutation can be distinctly discerned, demonstrating that multilevel switch-based gene circuit provides high detection specificity for single-nucleotide mutation (*n* = 4 technical replicates, bars represent mean ± SD, *P < 0.05, **P < 0.01).

For gene circuit-based assay, precise gene expression regulation is desirable to maximize the output while minimizing leaky expression that induces background noise. In this study, we designed a multilevel switch that simultaneously regulates the transcription and translation processes by the combination of STAR and THS and used the multilevel switch in the gene circuit. STAR would be turned on by the trigger RNA to allow the transcription of the downstream THS connected to the output enzyme. The hairpin structure of THS can be unwound by the trigger RNA, exposing the ribosome binding site and starting codon to activate translation of the downstream output (Fig. 2A). To evaluate the kinetics of activation, we used an MB-based reporter to detect the signal output level of gene circuit (Fig. S2).

Since the output signal of gene circuit is regulated by both the transcription and translation processes, more precise control of background noise may be achieved by the multilevel switch compared to regulation at single-level switch. To test this hypothesis, we compared single-level switch-based gene circuit with multilevel switch-based gene circuit for trigger RNA detection. When there is no trigger RNA, we observed that there exists evident background noise from single-level switch-based gene circuit regulated by either STAR or THS (Fig. 2B and C), while there is no detectable signal from multilevel switch-based gene circuit (Fig. 2D). The result was confirmed by the quantification of the signal, where the signal from multilevel switch-based gene circuit is close to the negative control group (no MB), indicating virtually no background noise (Fig. 2E). When activated by trigger RNA with the same concentration, gene circuits produced equivalent output signal (Fig. S2), indicating that the multilevel switch does not affect the maximum signal output. As expected, this multilevel switch improves the fluorescence signal (~4,000-fold over the control sample) with detection limit of 16.25 fM and increases the dynamic range (100 fM to 1 nM) (Fig. 2F). Meanwhile, the multilevel switch-based gene circuit is specific for single-nucleotide mutation detection (Fig. 2G). We designed 1 to 5 mutations in trigger RNA to demonstrate the accurate detection of multilevel switch-based gene circuit (Table S1). Notably, the fluorescence response produced by single-base mutation can be distinctly discerned, demonstrating the ultrahigh specificity of multilevel switch-based gene circuit. These results indicate that the designed multilevel switch that simultaneously regulates the transcription and translation processes can effectively suppress the background noise in the gene circuit.

### CATCH enables background-free ctDNA detection

**Fig. 3.**
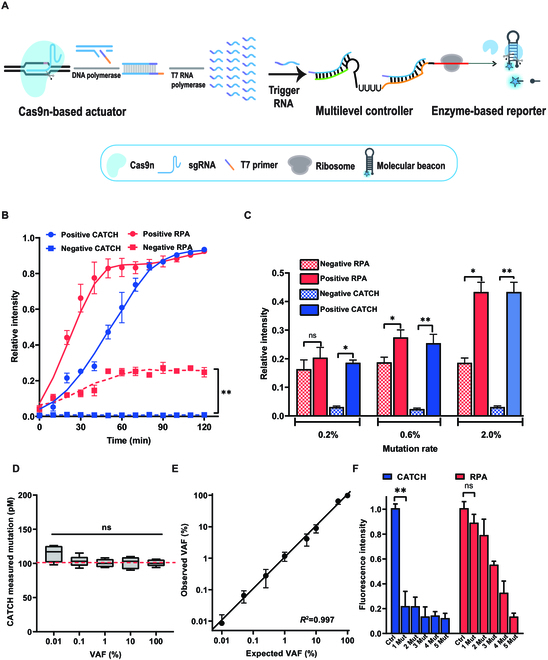
CATCH allows background-free ctDNA detection. (A) The scheme shows the ctDNA recognition process. Cas9n system recognizes ctDNA and generates a DNA–RNA hybrid and a nicking site. With the aid of DNA polymerase, a primer containing a 5′-T7 prompter sequence is introduced by strand displacement. Subsequently, the displaced DNA template can be exploited to produce trigger RNA through T7 RNA polymerase. Meanwhile, the trigger RNA unfolds the transcriptional and translational initiation sites in the multilevel switch. (B) Dynamic response curves of the CATCH and RPA-based assay were detected upon adding target DNA (0 and 1 pM). The fluorescence intensity of each sample is monitored. (C) Histograms show the capability of CATCH and RPA-based assay for detecting synthetic mutant DNA at different variant allele frequencies. (D) CATCH can stably detect the mutant allele concentration in samples with different concentrations of wild-type DNA. The red dotted line represents the actual content of mutant DNA. (E) CATCH is capable of highly sensitive detection of the 0.01% mutant allele DNA. Stability testing of the CATCH for detecting the mutant DNA spiked in 100 nM of the corresponding wild-type DNA with varied ratios (100% to 0.01%). (F) CATCH can be applied to detecting single-nucleotide mutation. Each concentration is 1.0 pM (*n* = 4 technical replicates, 2-tailed Student’s *t* test; bars represent mean ± SD; *P < 0.05; **P < 0.01).

Next, we integrated the multilevel switch-based gene circuit with the Cas9n recognizer and MB reporter to form the CATCH for ctDNA detection, which takes advantage of the high specificity of Cas9n in recognition. Cas9n can recognize ctDNA (Table S2) and only cleave one strand nick. Once Cas9n:small guide RNA (sgRNA) complex recognizes and cleaves nick, the following strand displacement synthesis extends at the nick, and the T7 primer binds to the 3′-end of nicked single-stranded DNA (ssDNA) to form a duplex, which acts as the template for T7 RNA polymerase-mediated transcription. Then, the polymerase generates trigger RNA along this template, which can activate multilevel switch-based gene circuit as the input to regulate the output restriction enzyme (Fig. 3A). The enzyme is then recorded by a nondenaturing polyacrylamide gel electrophoresis (PAGE) assay (Fig. S3). Upon addition of target DNA, the MB reporter is cleaved by the output restriction enzyme, generating a detectable fluorescence signal.

To overcome the potential incompatibility of different enzymes in CATCH, we first optimized the reaction components of CATCH in a tube (Fig. S4). We conducted a series of experiments to investigate the influence of the Cas9n:sgRNA ratio on the CRISPR/Cas9n system. We tested the ratios ranging from 8:1 to 1:8 and observed that the system reaches its maximum efficiency at a ratio of 1:4. For gene circuit expression, we evaluated the effect of plasmid concentration. We found that 5 pM is the optimal concentration for our experiment. Additionally, to determine the optimal temperature for the CATCH reaction, we incubated the CATCH mix at different temperatures (25, 30, 35, and 40 °C) in a bulk volume of 20 μl. We observed that the fluorescence signal reaches maximum intensity at 35 °C, indicating that this temperature is optimum for our assay. Finally, we optimized the reaction time for detection. By monitoring the fluorescence intensity over time, we found that after 90 min the fluorescence intensity reaches saturation, indicating that this time point provides sufficient time for the desired reaction to occur. Based on the specific recognition of Cas9n and the background-free output of multilevel switch-based gene circuit, CATCH could maximize the outcome while minimizing the background noise. We calculated the background signal of each assay as a percentage of its maximum signal. In comparison, CATCH displays a <0.01% relative background signal, while the RPA-based assay showed 190 times higher background signal. Besides, the RPA-based assay provides an SNR of 1.2 dB, while CATCH provides a much higher SNR of 11.5 dB (Fig. 3B), making it possible to distinguish low abundance targets. Next, we found that CATCH can detect single-nucleotide mutation-containing alleles in low abundance (<0.2%), while RPA-based assay shows poor performance in recognizing mutation DNA with high homology, which cannot accurately identify the target with a mutation rate below 0.5% (Fig. 3C).

To assess the accuracy of CATCH for low-abundance DNA detection, we applied CATCH to detection of mutant DNA diluted by a gradient of wild-type DNA. In mock ctDNA sample, CATCH can sensitively respond to low-concentration targets regardless of fixed mutant DNA concentration (0.1 nM; Fig. 3D) and fixed wild-type DNA concentration (100 nM) as reflected by the stable consistency (*R*^2^ > 0.99) (Fig. 3E). Moreover, the sensitivity of CATCH reaches up to 0.01%, which meets clinical demand [36]. The specificity of CATCH for single-nucleotide mutation is also superior to the RPA-based assay. In the presence of only one mutation, the signal of CATCH is significantly reduced, but the RPA-based assay shows much lower specificity to mutation change (Fig. 3F). These results indicate that CATCH offers comparable sensitivity and specificity for ctDNA detection with significantly higher SNR.

### Digital CATCH enables absolute ctDNA quantification

Given the ultralow concentration of ctDNA in biological fluids, highly sensitive detection technologies are demanding. Digital nucleic acid detection is an ultrasensitive method for nucleic acid detection where target concentrations can be absolutely quantified by Poisson statistics [10,37,38]. However, several limitations still need to be addressed. Such as the low signal differentiation between the positive and negative partitions and the false-positive partitions induced by nonspecific amplification. Due to the significantly reduced background noise and improved SNR, we hypothesized that CATCH can be integrated with a digital chip (i.e., digital CATCH) to circumvent these issues for absolute quantification of ctDNA.

**Fig. 4.**
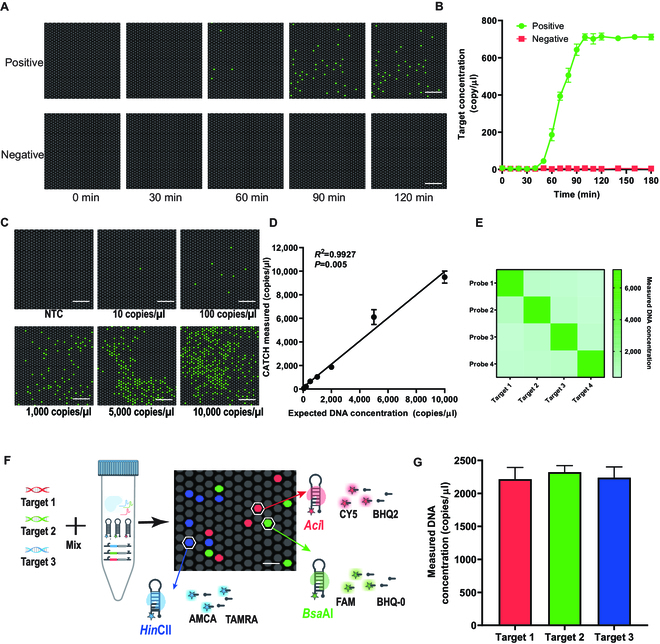
Digital CATCH for absolute and multiplex ctDNA quantification. (A) End-point fluorescence micrographs of the digital CATCH were captured at different time intervals at 35 °C. The positive group is with 2 × 10^3^ copies/μl of DNA, and the negative group is with no target added. Scale bars, 250 μm. Each micrograph is a representative region taken to cover about 1,100 partitions. (B) The percentage of positive partitions of digital CATCH was compared with digital RPA with various incubation times. The number of positive partitions is counted by setting the same threshold in the ImageJ software. (C) The puzzle shows representative end-point fluorescence images of various ctDNA concentrations for digital CATCH. Scale bars, 250 μm. (D) The scatter plot shows the linear relationship between the positive partitions and the target concentration. (E) Heat map analysis shows the orthogonal identification of 4 homologous DNAs. (F) The scheme shows the workflow of multiplex digital CATCH. The reaction mixture is prepared in one tube. After being partitioned into a microwell chip, each partition with target DNA generates detectable fluorescence signal. ctDNA concentrations can be quantified based on the proportion of positive partitions. (G) The results show parallel analysis of different ctDNAs on the same digital chip.

To determine if there is any unspecific signal output in digital CATCH, we integrated CATCH into a microwell chip containing 20,000 partitions and investigated the fluorescence signal change on the chip at various time intervals (i.e., positive signal) (Fig. 4A). We observed that the number of positive partitions is stable at 90 min and does not change over time for the positive group, while there are no positive partitions from the negative control group even after extending the reaction time to 150 min (Fig. 4B). For comparison, we also assessed digital RPA-based assay and observed clearly positive partitions at 10 min in the negative control group (Fig. S5), indicating that unspecific signal occurs.

Next, we assessed the absolute quantification capability of digital CATCH using serial dilutions of synthetic DNA of known concentrations (0 to 10^4^ copies/μl) and observed that the end-point fluorescence images display an apparent consistency between the target concentrations and the number of positive partitions (Fig. 4C). The measured concentrations are highly consistent with the input concentrations in the dynamic range from 10^1^ to 10^4^ copies/μl (*R*^2^ > 0.99) (Fig. 4D). Digital CATCH is well suitable for detecting rare mutant alleles in a large background of wild-type DNA. Our experiments demonstrated that digital CATCH can detect mutations as low as 0.01% variant allele frequency (VAF; Fig. S6A). Moreover, digital CATCH exhibits greater stability in detecting 0.01% VAF compared to digital PCR (Fig. S6B), highlighting its robustness and accuracy for quantification. Moreover, to validate the specificity of digital CATCH, we identified 4 different mutant DNAs (Table S3) with 2,000 copies/μl concentration and observed that digital CATCH allows signal output only when a specific mutant DNA is present (Fig. 4E). Besides, to assess the capability of digital CATCH for different DNA mutation detections in parallel (i.e., multiplex detection), we modified digital CATCH with 3 different sgRNAs for recognizing target DNA and outputting different restriction enzymes by corresponding gene circuits (Fig. 4F). Three MBs labeled with FAM, Cy5, and AMCA were prepared to produce green, red, and blue fluorescence on the digital chip after cleaving by corresponding output restriction enzymes, respectively (Table S4). The orthogonal testing result shows successful discrimination and ultralow cross-reactivity (Fig. S7). When there exist 3 target DNAs, 3 kinds of fluorescence can be clearly identified on the same chip (Fig. 4G). Next, we evaluated the potential of such multiplex digital CATCH to suppress cross-reactivity among different targets in one pot. For this, we spiked 2,000 copies/μl of different DNAs into one tube and observed that the multiplex assay retains no disturbance for their cognate target (Fig. S8). The results demonstrate that digital CATCH can achieve multiplex ctDNA mutation detection in one pot, which significantly reduces the detection cost and provides more detection information.

### Digital CATCH enables clinical detection of drug resistance gene mutations in NSCLC patient

Most cancer patients inevitably become drug resistant to first-line therapy [39], where drug resistance gene mutation detection is urgently needed for the patients with poor efficacy of targeted drugs for executing follow-up treatment [40]. Lately, ctDNA-assisted liquid biopsy has emerged as a promising alternative to traditional tissue biopsy for guiding medication due to its easy sampling and minimal invasion. Meanwhile, liquid biopsy brings the challenge in detection sensitivity due to the ultralow-abundance ctDNA in biological fluids. With the advantages of high sensitivity, high specificity, and wide dynamic range, digital CATCH holds great potential for monitoring drug resistance-related gene mutations. Here, we assessed the clinical capability of digital CATCH by detecting ctDNA from NSCLC patients' plasma.

**Fig. 5.**
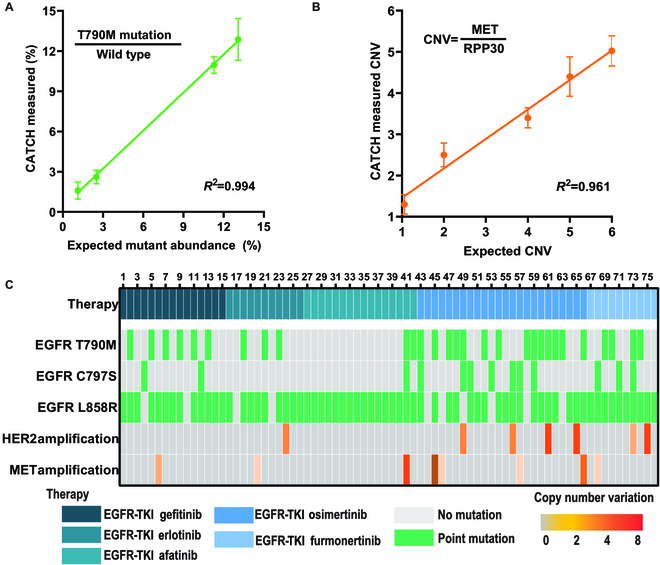
Digital CATCH enables clinical detection of drug resistance gene mutations in NSCLC patients. (A) Digital CATCH could detect single-nucleotide mutation in clinical samples. The plot shows the consistency between digital CATCH and expected value for EGFR T790M detection. (B) Digital CATCH could detect copy number variation (CNV) in clinical samples. The plot shows the consistency between digital CATCH and the expected value for MET amplification detection. (C) Digital CATCH achieves parallel detection from 76 clinical NSCLC patients' plasma samples. The heat maps show the comutation plot based on digital CATCH. Each column represents one patient. HER2/EIF2C1 > 2.0 was defined as HER2 amplification. MET/RPP30 > 2.2 was defined as MET amplification.

Epidermal growth factor receptor (EGFR) mutations are frequently observed in NSCLC patients [41], and acquired drug resistance can be target dependent. The L858R mutation and the exon 19 deletion account for approximately 85% of the EGFR mutations [42]. After treatment with the EGFR-tyrosine kinase inhibitors (TKIs), 55% of patients were resistant to EGFR-TKIs due to the T790M mutation [43]. While the third-generation EGFR-TKIs have shown positive effects in patients with the T790M mutation, drug resistance still occurs in patients with C797S mutation [44]. Moreover, human EGFR type 2 (HER2) amplification occurs in 8% to 13% of all cases. The proportion of patients with drug resistance due to *N*-methyl-*N*′-nitroso-guanidine human osteosarcoma transforming gene (MET) amplification is 5% to 9% [45]. These mutations are available as biomarkers of drug resistance [41]. The complexity of resistance mechanisms highlights the importance of repeated tumor biopsy for patients during disease progression [46]. Here, we applied our digital CATCH to testing these 5 clinically relevant gene mutations (i.e., EGFR L858R, EGFR T790M, EGFR C797S, HER2 amplification, and MET amplification). To verify that digital CATCH can directly obtain the mutation rate of the target gene, we first tested the reaction time of digital CATCH at different target concentrations and found that the time to reach the plateau phase is consistent for different target concentrations (Fig. S9). Thus, we can simultaneously detect mutant and wild-type ctDNA (Fig. 5A) and the ratio of amplified mutations (Fig. 5B) on one single chip.

Before testing with clinical samples, we synthesized artificial targets that precisely matched the sequence of 5 clinically relevant gene mutations. This allowed us to investigate the accuracy of CATCH at low abundances (Fig. S10). The results clearly demonstrated the capability of CATCH to detect mutations with VAF as low as 0.01% and target concentrations as low as 10 copies/μl. These findings highlight the suitability of digital CATCH for accurate quantification of these specific mutations in ctDNA.

To validate that digital CATCH is capable of detecting drug resistance gene mutations, we tested 76 plasma samples from 76 NSCLC patients treated with first-line EGFR-TKI, and compared the results from digital CATCH with gold-standard deep sequencing (CAPP-Seq) technique (Fig. 5C). The patients who were treated with EGFR-TKI have a proven EGFR mutation determined by deep sequencing. Among 67 patients with EGFR L858R mutation, 66 patients can be detected by our digital CATCH, showing a high sensitivity of 98.5%. Similarly, digital CATCH provides high sensitivity for the detection of T790M mutation (93.5%, 29 of 31), EGFR C797S mutation (91.7%, 11 of 12), MET amplification (88.9%, 8 of 9), and HER2 amplification (100%, 5 of 5). Notably, the consistency between digital CATCH and CAPP-Seq is very high with a κ value of 0.87.

To assess the clinical specificity of digital CATCH for disease monitoring, we compared different platforms for detecting ctDNA mutations. The detection capability of digital RPA-based assay is limited to some extent with low specificity (48.9%). In comparison, digital CATCH exhibits better detection capability with a sensitivity of 94.5% and a specificity of 98.8% (Table 1). In summary, CATCH enables highly sensitive and specific detection of drug resistance gene mutations in NSCLC patients.

## Discussion

In this study, we developed a detection assay termed CATCH for background-free and absolute quantification of ctDNA. A multilevel switch based on the combination of STAR and THS was designed and used in gene circuit to successfully eliminate background noise in ctDNA detection. Benefiting from such background-free and one-pot reaction features, CATCH was integrated with a digital microfluidic chip to form digital CATCH, enabling absolute quantification of ctDNA. Besides, modification of CATCH with multiple recognizers and reporters shows the potential for multiplex ctDNA detection. Finally, the clinical applicability of digital CATCH was verified by detecting 5 drug resistance-related gene mutations in 76 NSCLC patients, showing satisfying consistency with clinical standard methods.

CATCH is versatile for detecting ctDNAs. Compared with conventional NGS or PCR-based assays, CATCH can be implemented under a constant low temperature (35 °C), decreasing the requirements for peripheral devices (e.g., thermal cycler) and assay cost. Different from template amplification in PCR, CATCH’s high sensitivity relies on the T7 polymerase-mediated trigger RNA production and the highly efficient restriction endonuclease reaction, eliminating potential contamination and false-positive results in ctDNA detection [47]. Compared with digital PCR, digital CATCH showed comparable sensitivity in detecting rare mutant alleles. But for ultralow mutation rate detection (~0.01% VAF), digital CATCH shows superior repeatability. Moreover, digital CATCH does not require expensive and energy-consuming thermal cycler, thus being more suitable for point-of-care testing. Compared with isothermal amplification-based assays, CATCH efficiently eliminates background noise, resulting in superior detection sensitivity, specificity, and dynamic range. The detailed comparison of CATCH to these assays is shown in Table S5, which indicates that CATCH holds great potential as a promising alternative for ctDNA detection.

Gene circuit-based assays have been customized for highly portable and rapid nucleic acid detection [9,15,34,48–50]. Despite the advances, most gene circuit-based assays fail to provide sufficient nucleic acid detection specificity to reliably resolve the high background noise when no input exists [13,35]. Therefore, significant efforts have been carried out to address the background noise in the transcription [51], translation [52], and/or posttranslation [15,53] processes, where most research is based on single-level switch, leading to inevitable leaky expressions and instable output [54]. One way to address the challenge is to use multilevel switch to regulate gene expression in transcription and translation processes. For instance, 2 distinct riboswitches (i.e., antisense RNAs and THS) were proposed to reduce intracellular leakage expression in *Escherichia coli* [55] and cell-free expression system [35]. Although these multilevel switches are effective for regulating gene expression, the integration of multilevel switch to gene circuit for nucleic acid detection has not been explored yet. We demonstrate that the multilevel switch combining STAR and THS enables strict regulation of both the transcription and translation processes to minimize leakage, exhibiting ~4,000-fold in background repression. Moreover, CATCH is easy to operate and does not require additional expensive equipment. To our knowledge, this is the first report on using multilevel switch-based gene circuit for background-free nucleic acid detection.

CATCH provides accurate quantitative measurements even for low-concentration and low-frequency targets. The clear binary distribution of positive/negative signal observed in CATCH can be attributed to the effective functioning of the CRISPR/Cas9n system. Additionally, the implementation of a multilevel switch-based gene circuit ensures that the end point is less affected by background noise. CATCH demonstrates its suitability for detection of ctDNA in cancer patients, offering several advantages such as high accuracy, sensitivity, reproducibility, and the ability to perform absolute quantification. As a result, CATCH holds great promise in monitoring disease progression, assessing surgical outcomes, estimating prognosis, and diagnosing recurrence.

We have demonstrated that the gene circuit using the designed multilevel switch can effectively eliminate background noise, enabling background-free ctDNA detection by CATCH. Such multilevel switch design could be transplanted in other gene circuits for constructing assays with enhanced positive signal and reduced background signal. Although the multiplicity of CATCH has been prototyped, further investigations are necessary for extending CATCH for simultaneous detection of multiplex targets. Due to the simple one-pot reaction, CATCH could be further integrated with a portable device for implementing nucleic acid testing at point of care.

## Materials and Methods

### General materials

Unless otherwise noted, RNA and DNA oligonucleotides, primers, sgRNAs, and fluorescently labeled MBs were purchased from Tsingke Biotechnology Co. Ltd. (Xi’an, China) and used without further purification unless modification was required. Cell-free protein expression buffers were purchased from New England Biolabs (E6820, NEB, MA, USA). Assembly was performed using Golden Gate reaction with 4-base pair overhangs. Cas9n was purchased from Novoprotein Scientific Inc. (E366, Shanghai, China). Digital QuantStudio 3D chips were purchased from Thermo Fisher Scientific (Waltham, MA, USA). HiScribe T7 Quick High Yield RNA Synthesis Kit was obtained from NEB.

### Restriction enzyme expression for output

The restriction enzymes were designed and expressed in the cell-free gene expression system. Here, a 1.5-concentrated cell-free reaction increased the reaction kinetics [29]. Briefly, 20 μl of reactions was prepared using 10 μl of an optimized *E. coli* S30 lysate, 5 μl of NEB buffer 3.1 (pH 8.6), 0.5 μl of amino acid mix, 0.5 μl of methionine, 0.2 U of T7 RNA polymerase, and 1 μg of expression plasmids (Table S6). The reactions were incubated in a heating device. NEB buffer 3.1 remarkably enhanced the enzymatic activities as well as its detection sensitivities [56]. A fluorescence-based assay using MBs has been adapted from literature [57]. A fluorescence spectrophotometer was used to measure the fluorescence intensity. The excitation and emission wavelength of each channel were set to 346 nm and 442 nm (blue channel), 494 nm and 522 nm (green channel), as well as 646 nm and 664 nm (red channel), respectively. The bandwidth for all was set to 10 nm.

### CATCH

For bulk experiments, CATCH was performed in PCR strips at a final volume of 20 μl, consisting of different concentrations of target DNA, 100 nM T7 primer, 5 pM restriction enzyme expression plasmid with multilevel switch (Table S7), 160 nM sgRNA (Table S8), 40 nM Cas9n, 0.2 U of DNA polymerase, 0.2 U of T7 RNA polymerase, 0.1 U of ribonuclease inhibitor, 10 μl of optimized *E. coli* S30 lysate, 1.5 mM each amino acid, 0.2 mg/ml of tRNA, and 10 nM MBs. All bulk reactions were incubated at 35 °C for 90 min, and the fluorescence signals were continuously recorded with 1-min time interval.

To facilitate digital CATCH, the mixed CATCH reagent including 200 nM sgRNA and 0.1% Tween 20 was loaded into a commercial QuantStudio 3D Digital Chip. Excessive amounts of sgRNA could promote the formation of the Cas9n ternary complex, while Tween 20 could adjust the viscosity of the CATCH reaction mix compatible with the chip [58]. The chip was then heated at 35 °C for 90 min and transferred to a fluorescence microscope to acquire fluorescence micrograph. The subsequent data analysis was performed using GraphPad software, including statistical analysis, plotting, and data fitting.

### Clinical sample preparation

We obtained serum samples from 76 NSCLC patients at the First Affiliated Hospital, Xi’an Jiaotong University (Xi’an, Shaanxi, China). All patients were confirmed by histopathology. The cfDNA samples were extracted using the QIAamp circulating nucleic acid kit. Ten milliliters of plasma was mixed with 300 μl of magnetic bead suspension, 550 μl of proteinase K, and 1,500 μl of bead binding buffer in a 15-ml tube. The mixture was incubated for 10 min at room temperature with end-over-end shaking. Next, the tube was placed into a magnetic rack and left for at least 1 min until the solution became clear. After elution from the beads, the supernatant was pipetted into a QIAamp UCP MinElute column and centrifuged for 1 min at 1,400 rpm. Finally, 30 μl of water was pipetted into the center of the membrane. To elute the nucleic acids, the sample was centrifuged at 20,000 rpm for 1 min. All plasma samples were analyzed by CAPP-Seq by commercial sequencing institutions, the manufacturer as described previously [59]. The presence of ctDNA is identifiable in all patients selected in this work. This clinical study was approved by the Ethics Committee at the First Affiliated Hospital of Xi’an Jiaotong University.

### Digital PCR

A total 15 μl of PCR was prepared and added to the digital chip, which includes 2× droplet digital PCR supermix, 100 nM primers, and 1 μM fluorescent probes. The thermal cycling program is as follows: 95 °C for 10 min, 35 cycles of 95 °C for 30 s and 60 °C for 1 min, 95 °C for 15 min, and hold at 4 °C. The heating and cooling rates are 2 °C/s for all steps.

### RPA

The TwistAmp Liquid Basic Kit was used according to the instructions. In brief, the total reaction volume was 20 μl, including 120 nM primer, 10 mM deoxynucleotide triphosphates, and 8 mM magnesium acetate. RPA reactions were incubated at 37 °C for 1 h.

### Chip image analysis

The 3-color intensity vectors for each microwell were projected onto the 2-dimensional image unit, and the density of each color was identified by ImageJ. The mapping of the fluorescence of each color was determined by graphics integration.

### Statistics

Statistical analysis, plotting, and data fitting were performed with Excel and GraphPad Prism 8. Two-tailed *t* tests and 2-sided Fisher’s exact tests were used for the significance test. One-way analysis of variance and post hoc Tukey's multiple comparison test were performed. The data were presented as mean ± SD. Statistical significance was assumed at *P* < 0.05 (*P < 0.05, **P < 0.01).

For the background signal and SNR calculation, the end-point fluorescence intensity at 90 min (*R*_t_) was divided by the initial fluorescence intensity at 1 min (*R*_b_). The quantitative curve was determined by the ratio (*R*_t_ − *R*_b_)/*c*. *c*, the analyte concentration or its logarithm, log *c*. The SNR (dB) was calculated using the following equation:

$$\mathrm{SNR}=20\cdot \log_{10}\frac{\log_{10}\left(R_{t}/R_{b}\right)}{2\cdot \log_{10}\left(\sigma \right)}$$

where σ is the geometric SD of the distribution for *R*_b_.

## Data Availability

Data are available from the corresponding authors upon request.
