# Supplementary material for: Synthetic Gene Circuit-Based Assay with Multilevel Switch Enables Background-Free and Absolute Quantification of Circulating Tumor DNA
Source: Research (Wash D C). 2023 Oct 2;6:0217. doi: 10.34133/research.0217 (PMC10543738; doi:10.34133/research.0217)
Supplement: Supplementary 1 — Figs. S1 to S10 Tables S1 to S8 [file research.0217.f1.docx]

**Supplementary Information**

**Supplementary data figures:**


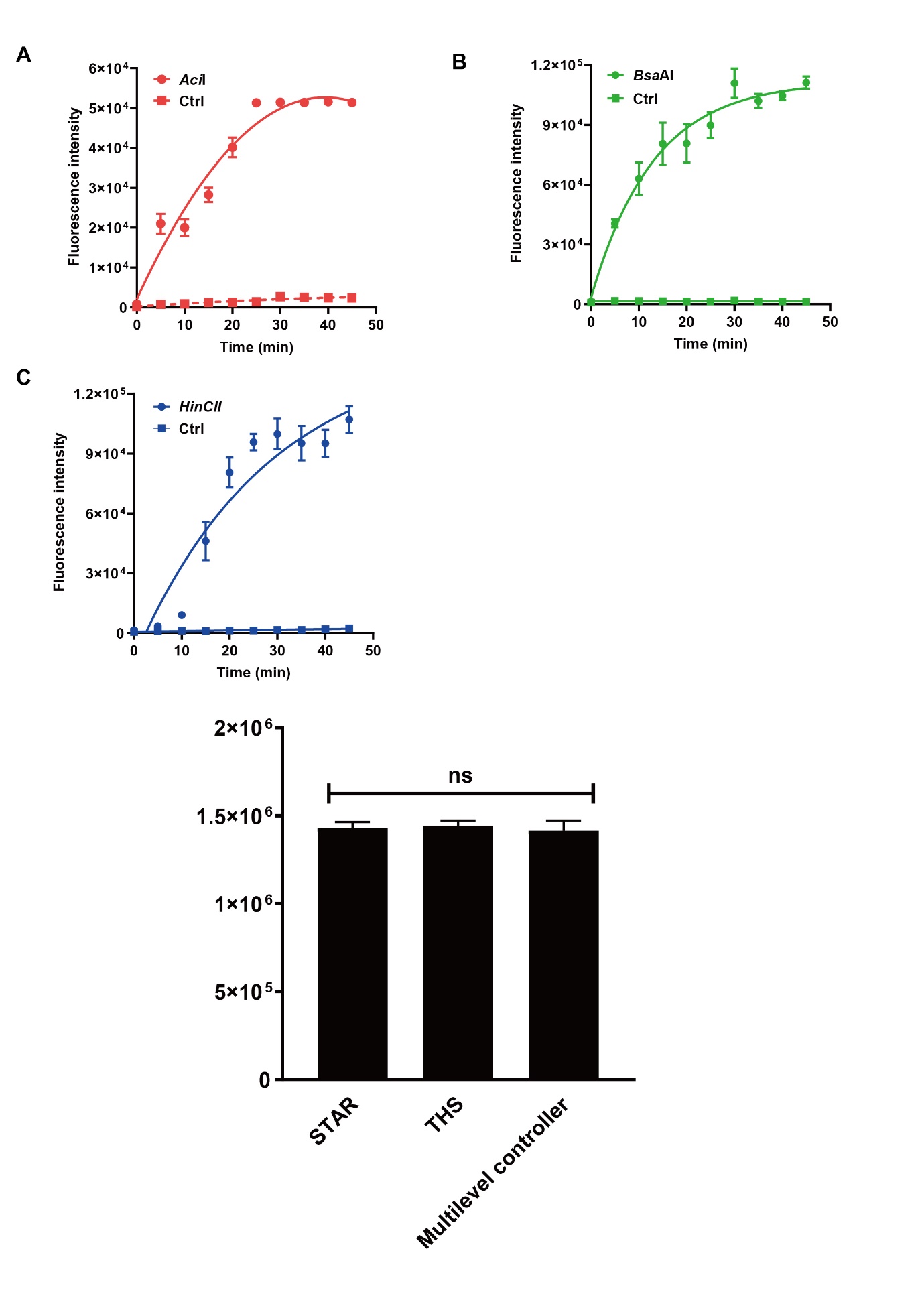


**Supplementary Figure S1.** The kinetics of three restriction enzymes in MB cleavage assays.


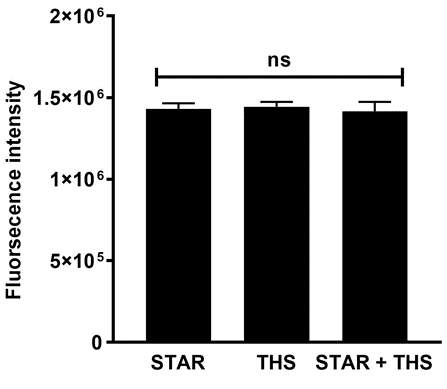


**Supplementary Figure S2.** When activated by the 10 nM of trigger RNA, different switch-based gene circuits produce approximate output signals.


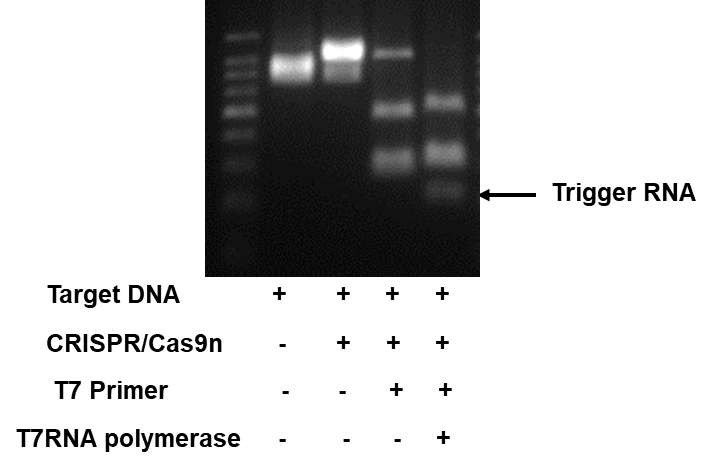


**Supplementary Figure S3.** Schematic represented and verified the formation of trigger RNA. PAGE confirmed successful strand elongation of the T7 template and trigger RNA. Without DNA polymerase (lane 2), we observed only one band, the same as lane 1. When DNA polymerase was added (lane 3), a new band appears, suggesting that the target was cleaved and the displaced strand has been generated. A new band was observed in lane 4 compared with the band in lane 3 when T7 RNA polymerase was added, suggesting trigger RNA was synthesized.


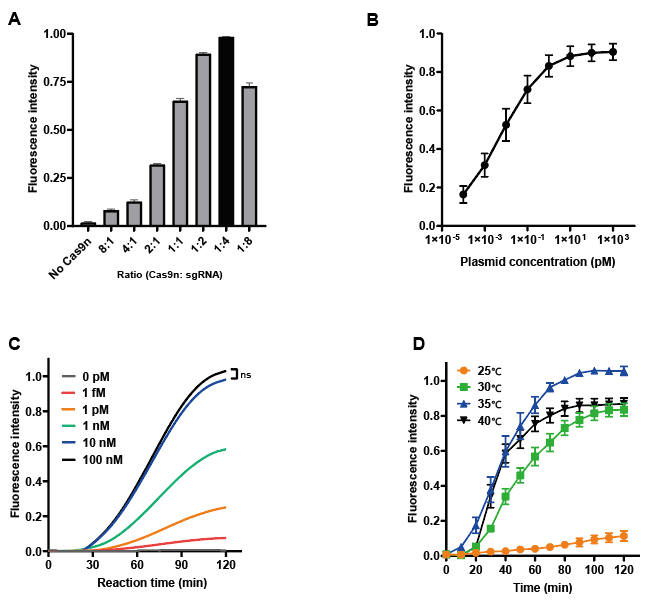


**Supplementary Figure S4.** Optimization of CATCH reaction conditions. **(A)** The concentration ratio of Cas9n and sgRNA. The ratio of 1:4 is selected for Cas9n: sgRNA. **(B)** Optimization of the plasmid concentration. When the concentration reaches 5 pM, the fluorescence intensity reaches plateau. **(C)** Optimization of the MB concentration. The fluorescence intensity increases gradually to 10 nM. **(D)** CATCH is conducted in one-pot, and the fluorescence signal was saturated at 90 min after incubation at 35°C.

**
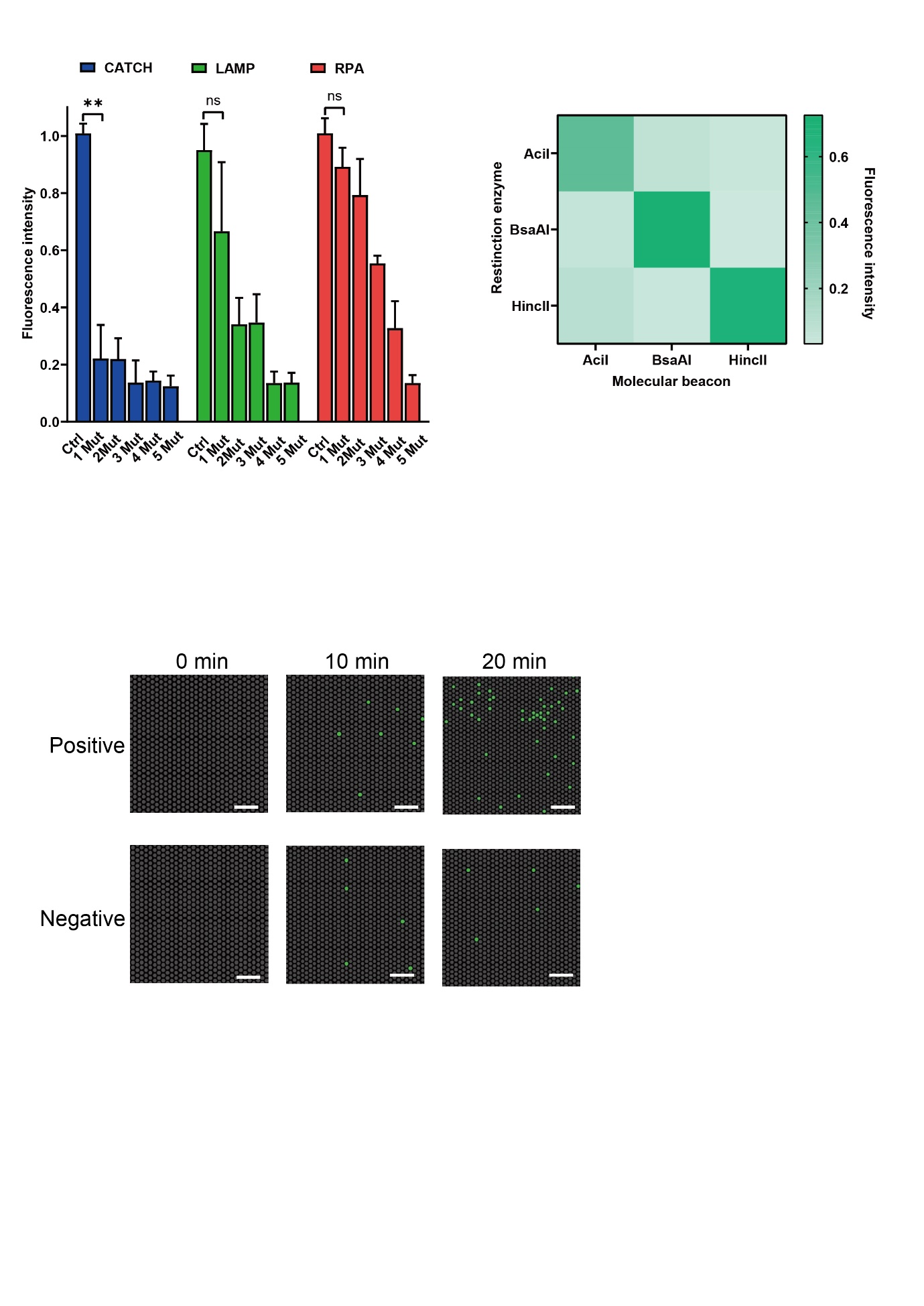
**

**Supplementary Figure S5.** Endpoint fluorescence micrographs of the chip for digital RPA-based assay with various incubation time at 37 °C. Positive, the reaction with 2 × 10^3^ copies/μL DNA. Negative, non-target. Scale bars are 250 μm.


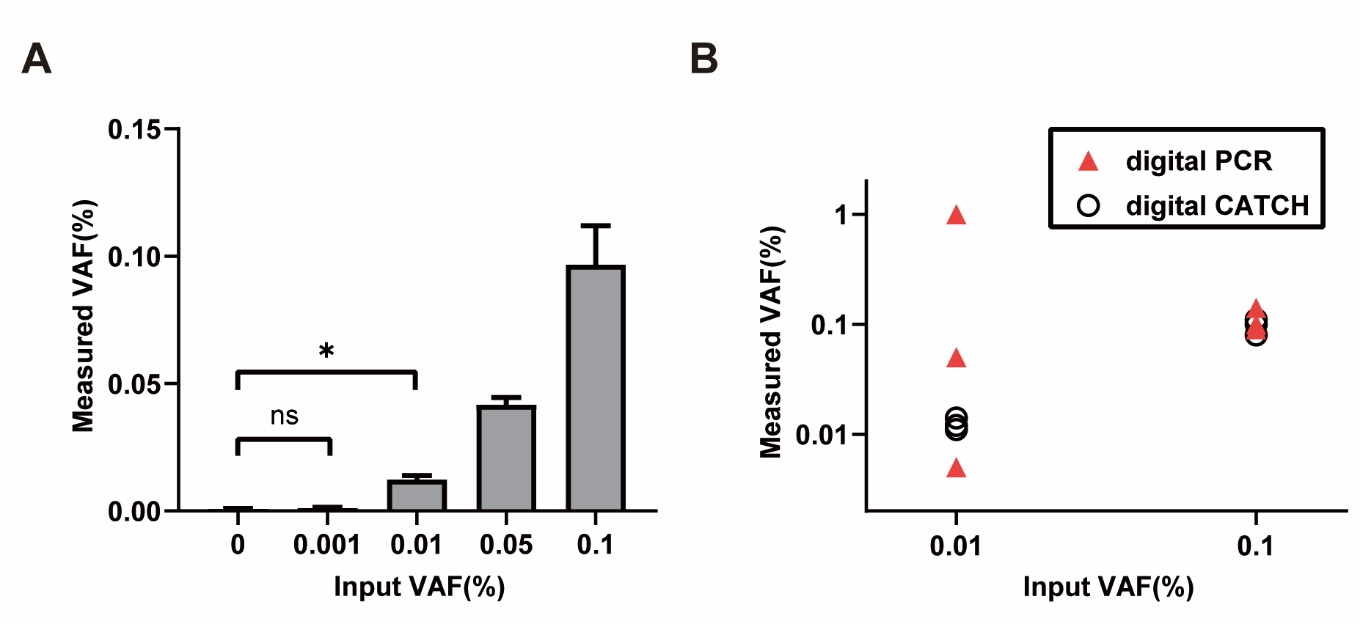


**Supplementary Figure S6.** Analysis of digital CATCH performance in detecting rare mutant alleles of synthetic fragments. (A) Evaluation of the digital CATCH's ability to detect different mutation frequencies. (B) Analytical comparison in 0.01% to 0.1% VAF between digital CATCH and digital PCR.


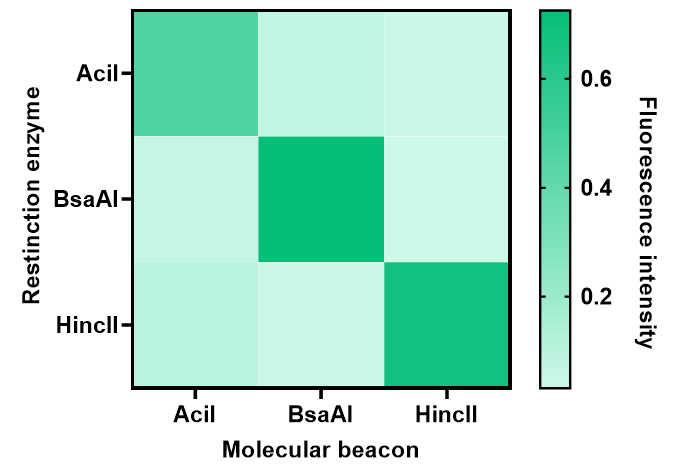


**Supplementary Figure S7.** Heat map of the specific restriction enzyme activity.


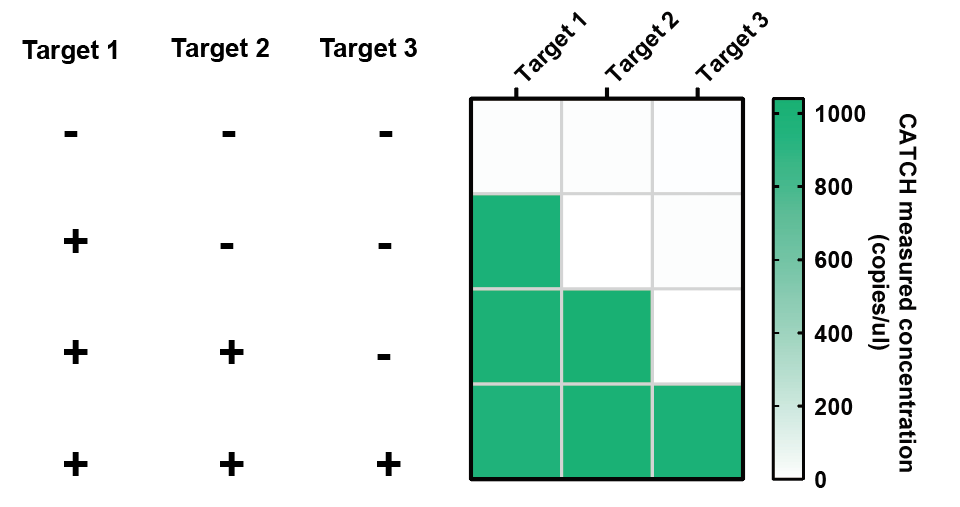


**Supplementary Figure S8.** In-sample multiplex detection of different target DNA. CATCH could distinguish all combinations of targets.


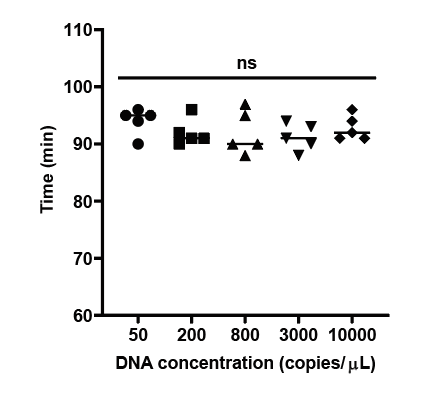


**Supplementary Figure S9.** Comparison of the time for different DNA concentrations to reach the plateau.


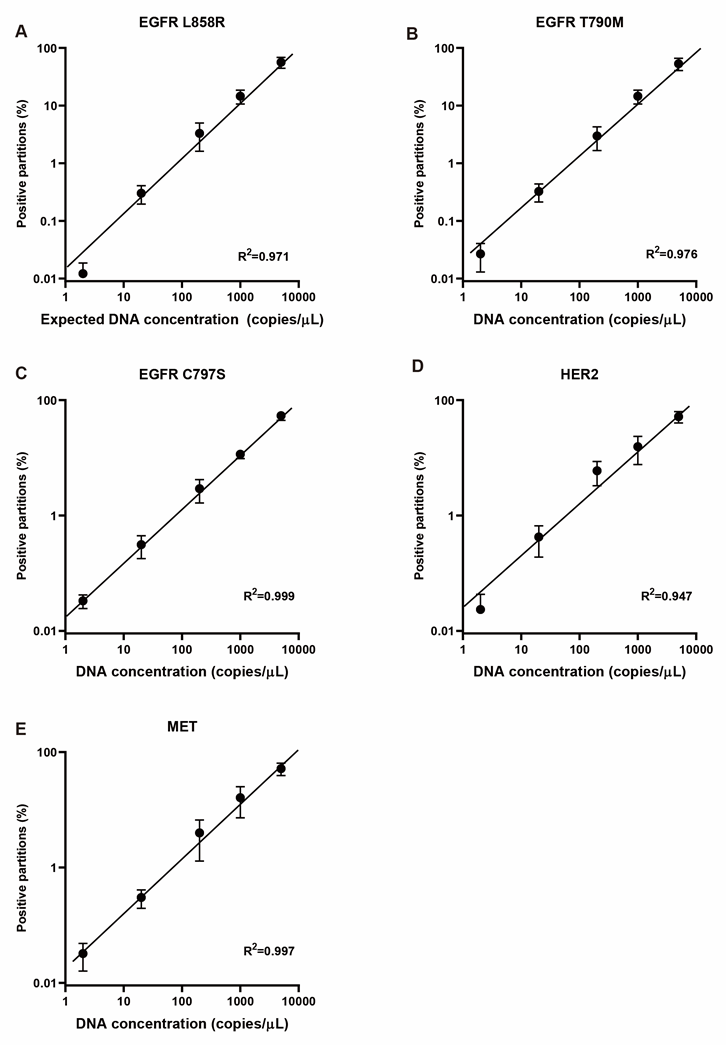


**Supplementary Figure S10**. The linear relationship between the percentage of positive partitions in digital chip and the concentration of targets for (A) EGFR L858R, (B) EGFR T790M, (C) EGFR C797S, (D) HER 2, (E) MET detection. For each testing, three independent assays (n = 3) were implemented. Error bars represent the means ± s.d. from replicates.

**Table S1. Sequence of trigger RNA used in this study**

| Name | Sequence, 5’-3’ | Used in |
| --- | --- | --- |
| Trigger RNA | ACGGCUGGAGACACUGAACAUCCCGGUGGUCG | Fig. 2 |
| Mutant trigger RNA 1 | ACGGCUGGAGACCCUGAACAUCCCGGUGGUCG | Fig. 2 |
| Mutant trigger RNA 2 | ACGGCUGGAGACCGUGAACAUCCCGGUGGUCG | Fig. 2 |
| Mutant trigger RNA 3 | ACGGCUGGAGACCGCGAACAUCCCGGUGGUCG | Fig. 2 |
| Mutant trigger RNA 4 | ACGGCUGGAGACCGCCAACAUCCCGGUGGUCG | Fig. 2 |
| Mutant trigger RNA 5 | ACGGCUGGAGACCGCCUACAUCCCGGUGGUCG | Fig. 2 |

**Table S2. Sequence of target DNA used in this study**

| Name | Sequence, 5’-3’ | Used in |
| --- | --- | --- |
| Target DNA | CATTTACCAGTAAATCTGGTGGCGTGTTATAGTTA  TGAACAGAGGAGACATAACATGAACACGCCAAAC | Fig. 3 |
| Mutant target DNA 1 | CATTTACCAGTAAATCTGGTGGAGTGTTATAGTTA  TGAACAGAGGAGACATAACATGAACACGCCAAAC | Fig. 3F |
| Mutant target DNA 2 | CATTTACCAGTAAATCTGGTGGAGAGTTATAGTTA  TGAACAGAGGAGACATAACATGAACACGCCAAAC | Fig. 3F |
| Mutant target DNA 3 | CATTTACCAGTAAATCTGGTGGAGAGATATAGTTA  TGAACAGAGGAGACATAACATGAACACGCCAAAC | Fig. 3F |
| Mutant target DNA 4 | CATTTACCAGTAAATCTGGTGGAGAGATCTAGTTA  TGAACAGAGGAGACATAACATGAACACGCCAAAC | Fig. 3F |
| Mutant target DNA 5 | CATTTACCAGTAAATCTGGTGGAGAGATCTCGTTA  TGAACAGAGGAGACATAACATGAACACGCCAAAC | Fig. 3F |

**Table S3. Target DNA for multiplex detection**

| Name | DNA sequence, 5’-3’ | Used in |
| --- | --- | --- |
| Target 1 | GCGCTAATACGACTCACTATAGGGAAAATG  TGCTGGATACGCTGGATCGCTTGGGCGTAG  TTATAGTTATGAACAGAGGAGACATAACA  TGAACTACGCCAACGTAGTTAATTTAGCAG  CAGCACAAAAGACCATGATTGCGCTAATACG  ACTCACTATAGGGAGACACCAATC | Fig. 4E |
| Target 2 | GCGCTAATACGACTCACTATAGGGACAAAA  AAGCCACTGCACCAAAAGACACCATCTATCG  TTATAGTTATGAACAGAGGAGACATAACATG  AACGATAGAAACATCGTTAATTTAGCAGCAG  CACAAAAGACCATGATT | Fig. 4E |
| Target 3 | GCGCTAATACGACTCACTATAGGGAGACACCA  ATCCATTTACCAGTAAATCTGGTGGCGTGTTAT  AGTTATGAACAGAGGAGACATAACATGAACA  CGCCAAACCGTGTTAATTTAGCAGCAGCACAA  AAGACCATGATT | Fig. 4E |

**Table S4. Molecular beacon for restriction enzyme**

| Name | Sequence, 5’-3’ | Used in |
| --- | --- | --- |
| MB for AciI | CCCCTCCGCGCGCCCGGTTTTTCCGGGCGCGCGGAGGGG | Fig. 2, 3, 4, 5 |
| MB for BsaAI | CCCCTTACGTGGGGGCCGGTTTTTCCGGCCCCCACGTAAGGGG | Fig. 2, 3, 4, 5 |
| MB for HincII | CCCCTGTTGACCGGGCCGGTTTTTCCGGCCCGGTCAACAGGGG | Fig. 2, 3, 4, 5 |

**Table S5. Detailed comparison with ctDNA detection technologies.**

|  | NGS | qPCR | RPA | THS | CATCH |
| --- | --- | --- | --- | --- | --- |
| Cost (USD) | 23 | 2 | 1 | 0.8 | 0.85 |
| Sensitivity | aM | aM | aM | fM | aM |
| Time | 16 h | 3 h | 1 h | 1.5 h | 1.5 h |
| Background-free | Yes | Yes | No | No | Yes |

**Table S6. Codon optimized sequences in restriction enzymes expression plasmids.**

| Enzyme | Codon Optimized Sequence |
| --- | --- |
| AciI | GCGAATTAATACGACTCACTATAGGGGAATTGTGAGCCGATAACAATTCCCCTCTAGAAATAATTTTGTTTAACTTTAAGAAGGAGATATACGATGGGCAGCAGCCATCATCATCATCATCACAGCTCTGGCATCGAAGGGCGCAGTGGGGGAGGAGGGTCCATGAATGAGCACATCAAGGGCTCTAATAGTCACGGCAACTCAAATGAGTTGGAATTGGTTTACGCCTTTGACGGTAAGAAGGTCAAAGATTTGAATACCAATCTTAAGAACTTTGTCCAATTCATTGCTAATGACAACAATATTAAGATTAATAATGATACTAAGCTGTTCGCGAAATACGTAAGTAATAATAAGCTGAAACAGGACTTTATTGTTTCATTCAATGAACGTGACTTCTATATCAGTTTGAAGATGGGGAGTGGTAACTCTGTGCATCAGGAGCCCATTGAAGATTTCATCAAATACTTGAACACAAACTATGAAGTAACGGAGAAGATTTGCAATGATCTTCGCTTTTTTATCTGGGCAGACGGTACATTAGACGGCAAGGGGAAGTTCGAGAATCGCTTCGATGCTCGCTATTTTAAGAAGAACTATCCCGAGAAGCGTCGCGAATTGTTGCAGTTTTTCGAGAAGAACAAAGTCGAGCTGATTAAGCATTTTATGTTCGTTGGTAAACATAATTCGCGCGTTGATTATCTGTATCACGGAACTACTTCCAACGGAGCCTGGATGTCAACTAAGCAAATTATTGATTATAATATCCAAAACCAGATCGACACGAACAAGGGTAACAGTCCAACATTGTCTGTTGGACGTATGAGCATCCAAGCGTGGAACGTCGCCAAATCCGGATCTGAATCAGCCGAGAAGAAACGTGGTGAAATTCAAGTGAAATACGGAAAGTTAAAGGAAGACTTCAAAGAGGTACTTAAACTGAATAGTAGCAATAAAGGAACTTTATTTGGAGACCACGAGGAGTTTGACATCTCCGGAACATTGAACAAGAATAAAAATCACTTTTACTGGAAGATGATCGCGCGCGACTTAAACTTGAATCAAGAAGAATTAAATAATTTATATGTGGTCCGTGTAAGCTCGAAAGTTATGAGCAGTTTATCTAAAAAGAAGGTACTTCCCAAATCTGACGCCTACATTATTCGCGCAGACTTGTCAAAATCATTCCTGCTTAGTAAGGAGTACAAACTTAGCGAAGACGATCTTGTTGGCATCATTTACAAGAAAGTTGGACGCTCGGGAATTTCGGTAAAACGCGCAGATTCCAAAAAATACACCATTGTAAAATTAACAGTTGCGAGCTTTGAAAAGTGCTTTGAAATCGAACCTGAAATTAAGAAAATTATCGCCGGTTTACTTTTATACTCGAAGGAAAAAGACATGTACAAGAATTTAGAAATTCTGAACAAGATCGGTATTAGCGAGTTGGAGCTTATCAACTACACAAACCGTTTCATTGTCGACAAGGTAATTTCATGCAATGACCCGAAGAATGTAGATATTATTCGTTCTACAATGCAGGAGCGTACACGTACCCTGATTGAGAAGAATCTTGAGATCAAAAAAGCATTGTTCATGGGTGAGGGTTGGTACGAGGAGCCATACTGCATCAACTATATTTTTAAGGATGGAAAATTATCGAACGACGTGTTTTCCGAGTACATCATTACTACGGGCAGTGGTCGTTCAAAGGGGAATTATACGATTGCCTTGAAACCAAAGAGTGGAGGAGGAGGCAGTGAAAACTTATACTTCCAGAGCAGCTCTGGCGACTACAAAGATGATGATGATAAATAACTAGCATAACCCCTCTCTAAACGGAGGGGTTT |
|  |  |
| BsaAI | GCGAATTAATACGACTCACTATAGGGGAATTGTGAGCCGATAACAATTCCCCTCTAGAAATAATTTTGTTTAACTTTAAGAAGGAGATATACGATGGGCAGCAGCCATCATCATCATCATCACAGCTCTGGCATCGAAGGGCGCAGTGGGGGAGGAGGGTCCATGTACAATTATTTATTAAACGAAAATGCAGATATAATTTATGATGGAAAGGTTATACTCACAAAAGAACAAGTTGTTGAAGCAATTATTATAACAAATACAAACTTAAAGAAACTTAATGACATTACGAAAGAGTCTGGTGTCGAAGTGTTTGAAGCATTAGGAATGAGAAACCTAAGTGGTTTTATTGGTGAGTTTTTCGTAAGCAGTCTCGAACAAGTATCAAATAAGAACTTAGTTAAAAATCCACATCAAGATGGATACCCAGATTTGTTGCTCGTAGATTCTCCTAAAGCTGCCTCATACTTTAATTCAATAGTCGAAATAGTTGATGGAAAATTATATCCAAAAGAAAAAAGTCTGTTTAGCCCATTTAAATATGGTGGATTAGAGGTAAAAGCCACTTGTGGTTCTACACCTTCAGCAAAAGTTATGCCTAAGCCATTGATTGGCGAGCAGAGAATTCACATCTTAACTGGATTAGATTGGAAGGCCCATCATAGAGGTACTAACAATCTAATAGGAATATATTGGGATTTTTTAGATGAGTTACCAACCATTTGCGCTGTATTTTATAGAAACGACCTAACCGAAGATGATTGGGGAAAAATTGTTCGCCCTAAAGAAGGTGGGGGAAGAACCACAAGTGTATCCATTATGAACTCAAAGGGTGTCAAAAAAATGTGCAAGAACTGGATTGCTATTATTGATAACGAAGATTATATAAACGCATTTTCTAATAAAAAATGGATAGGATATAATGTAAAAAACTCATCAAATAGTGGAGGAGGAGGCAGTGAAAACTTATACTTCCAGAGCAGCTCTGGCGACTACAAAGATGATGATGATAAATAACTAGCATAACCCCTCTCTAAACGGAGGGGTTT |
|  |  |
| HincII | GCGAATTAATACGACTCACTATAGGGGAATTGTGAGCCGATAACAATTCCCCTCTAGAAATAATTTTGTTTAACTTTAAGAAGGAGATATACGATGGGCAGCAGCCATCATCATCATCATCACAGCTCTGGCATCGAAGGGCGCAGTGGGGGAGGAGGGTCCATGAGTTTCATAAAACCTATTTATCAGGATATTAATTCAATATTAATCGGGCAAAAAGTGAAACGTCCTAAATCAGGTACTCTGTCAGGTCATGCTGCAGGGGAACCATTTGAAAAATTAGTATATAAGTTTTTGAAAGAAAACCTGTCAGATTTAACATTTAAGCAATATGAATATCTTAATGATTTATTTATGAAGAACCCTGCGATAATTGGACATGAAGCTAGATATAAATTATTTAATTCTCCAACATTGCTTTTTTTGTTAAGTAGAGGTAAAGCTGCAACTGAAAATTGGAGCATAGAAAATTTATTTGAGGAGAAACAAAATGATACTGCAGATATTTTATTAGTAAAGGATCAGTTCTATGAATTGTTAGATGTCAAAACAAGAAATATTAGTAAATCAGCTCAAGCACCCAATATTATTTCAGCATATAAATTAGCTCAGACGTGTGCAAAAATGATTGATAATAAAGAATTTGATTTATTTGATATTAATTATTTAGAAGTAGACTGGGAACTTAATGGTGAAGATCTAGTTTGTGTATCTACTTCTTTTGCTGAACTATTTAAATCTGAACCTAGTGAACTATATATTAACTGGGCTGCAGCTATGCAAATTCAGTTTCATGTAAGAGATTTAGATCAGGGGTTTAATGGAACTAGAGAAGAATGGGCAAAATCTTATCTAAAACATTTTGTTACTCAAGCAGAGCAAAGAGCCATATCTATGATAGATAAGTTTGTTAAGCCATTTAAGAAATATATACTTAGTGGAGGAGGAGGCAGTGAAAACTTATACTTCCAGAGCAGCTCTGGCGACTACAAAGATGATGATGATAAATAACTAGCATAACCCCTCTCTAAACGGAGGGGTTT |

The DNA sequences for these enzymes were obtained from online databases (NCBI) and REBASE data banks (http://rebase.neb.com/rebase/rebase.html), and codon optimized for cell-free expression using the Optimization Tool (idtdna).

**Table S7. sgRNA for mutation site in drug resistance genes**

| Name | Spacer sequence in sgRNA | Used in |
| --- | --- | --- |
| EGFR L858R | UCAAGAUCACAGAUUUUGGG | Fig. 5 |
| EGFR T790M | AUCAUGCAGCUCAUGCCCUU | Fig. 5 |
| EGFR C797S | GCCAGCGTGGACAACCCCCA | Fig. 5 |
| HER2 | AUGGCUGGUGUGGGCUCCCC | Fig. 5 |
| MET | GUGCAUGUUUGUGCCUGUGUCC | Fig. 5 |

**Table S8. Sequence of multilevel switch for gene mutation site in EGFR-TKI resistance**

| Name | Sequence in plasmid, 5’-3’ | Used in |
| --- | --- | --- |
| EGFR L858R | AATTGACTCTCTTCCGGGCGCTATCATGCCATACCGCGAAAGGTTTT  GCGCCATTCGATGGTGTCCGGGATCTCGACGCTCTCCCTTATGAAGT  CTAATGGGCGCTGCTCCTAACTGTCGCGCTAATACGACTCACCTGTT  ATAGGGTATAAGTAAATCGCTTGATGTCGTTAAACAGAGGAGATAAC  GAATGACAGCAAGCAACCTGGCGGCAGCGCAAAAGATGCGTAAA | Fig. 5 |
| EGFR T790M | AATTGACTCTCTTCCGGGCGCTATCATGCCATACCGCGAAAGGTTT  TGCGCCATTCGATGGTGTCCGGGATCTCGACGCTCTCCCTTATGAA  GTCTAACGCTGCTCTGGGCTAACTGTCGCGCTAATACGACTCACTA  TAGGGCTTACTACTTTGACACCTGATTCTGACACGATAACAGAACA  GAGGAGATATCGTATGAGAATCAGGAACCTGGCGGCAGCGCAAAA  GATGCGTAAA | Fig. 5 |
| EGFR C797S | AATTGACTCTCTTCCGGGCGCTATCATGCCATACCGCGAAAGGTTT  TGCGCCATTCGATGGTGTCCGGGATCTCGACGCTCTCCCTTATGAA  GTCTAACGCTGCTCTGGGCTAACTGTCGCGCTAATACGACTCACTA  TAGGGTGATGGAATAAGGCTGTGTATATGATGTTAGACAGAGGAG  ATAACATATGATACACAGCAACCTGGCGGCAGCGCAAAAGATGC  GTAAA | Fig. 5 |
| HER2 | AATTGACTCTCTTCCGGGCGCTATCATGCCATACCGCGAAAGGTTT  TGCGCCATTCGATGGTGTCCGGGATCTCGACGCTCTCCCTTATGAA  GTCTAACGCTGCTCTGGGCTAACTGTCGCGCTAATACGACTCACTA  TAGGGAGTTTGATTACATTGTCGTTTAGTTTAGTGATACATAAACAG  AGGAGATATCACATGACTAAACGAAACCTGGCGGCAGCGCAAAAG  ATGCGTAAA | Fig. 5 |
| MET | GCGCTAATACGACTCACTATAGGGAAAATGTGCTGGATACGCTGGAT  CGCTTGGGCGTAGTTATAGTTATGAACAGAGGAGACATAACATGAA  CTACGCCAACGTAGTTAATTTAGCAGCAGCACAAAAGACCATGATT  CGTGTATGTTCAGCTATCTGGGCGCGGATGAGTATGATGTCGATACC  GCCAAATACCAAGAAAATGTGCTGGATACGCTGGATCGCTTGGGCG  TAAGTATCTTGTGGCGTGATAATAATTCGGACTCAAAAGGCGTGATG  GATAAGCTGCCAAAAGCGCAATTTGCCGATTATAAACCCTATAGTGA  GTCGTATTAGCGC | Fig. 5 |
